# Synchronous Sound Recognition and Energy Harvesting by Flexible Piezoelectric PLLA/VB_2_ Composites

**DOI:** 10.3390/polym16081071

**Published:** 2024-04-11

**Authors:** Qian Zhang, Qiang Liu, Weidong Xue, Yong Xiang, Xiaoran Hu

**Affiliations:** 1 School of Materials and Energy, University of Electronic Science and Technology of China, Chengdu 611731, China; zq@uestc.edu.cn (Q.Z.); qiangrye@163.com (Q.L.); jerryliu158@163.com (W.X.); xyg@uestc.edu.cn (Y.X.); 2 Tianfu Jiangxi Laboratory, Chengdu 611731, China

**Keywords:** polylactide, Vitamin B_2_, sound recognition, piezoelectricity, energy harvesting

## Abstract

In the present study, poling−free PLLA/VB_2_ piezoelectric composites are fabricated to achieve synchronous sound recognition and energy harvesting. The addition of VB_2_ can interact with PLLA by intermolecular hydrogen bonding, inducing the dipole orientation of C=O in PLLA. Meanwhile, VB_2_ can promote crystallization of PLLA through heterogeneous nucleation. The combination of the two strategies significantly improves the piezoelectric performance of PLLA/VB_2_ composites. The PLLA/VB_2_ can detect the sound frequency with an accuracy of 0.1% in the range of 0–20 kHz to recognize characteristic sounds from a specific source. PLLA/VB_2_ can also convert sound into electrical energy synchronously with an energy density of 0.2 W/cm^−3^ to power up LEDs. Therefore, PLLA/VB_2_ shows great potential in the field of information and energy synchronous collection.

## 1. Introduction

The increasing demand for energy supplies of portable terminals and wearable devices has prompted research on ambient energy harvesting [1,2,3,4,5]. The ambient energy harvesting technology allows to collect and convert ambient mechanical, thermal, and electromagnetic energy to electricity to achieve a clean and sustainable energy supply [6,7,8,9,10]. Among them, harvesting of ambient mechanical energy is the most investigated due to its wide availability and ease of collection. By using piezoelectric generators (PEGs), the low and irregular mechanical energy can be efficiently converted to electrical power due to the piezoelectric mechanism [11,12,13].

Currently, most research on harvesting ambient mechanical energy is related to human body motions such as breathing, walking and running due to its high power density [14,15,16,17,18]. However, such an approach is limited by specific application scenarios. In contrast, one of ambient mechanical energy or sound energy is ignored due to its relatively low power density even though they are widely available without application scenarios limitations. As PEG is prepared by materials with piezoelectric properties, the efficiency of electricity generation of PEG is strongly dependent on the piezoelectricity of the material used. This means materials with higher piezoelectricity are of great concern when designing PEG for harvesting sound energy.

Piezoelectric polymers, such as poly (vinylidene fluoride) (PVDF), offer natural flexibility and a low weight that makes them suitable for energy harvesting in portable and wearable applications compared to inorganic piezoelectric materials. PVDF has a high piezoelectric output for mechanical energy harvesting [19,20], but it requires additional poling to form the piezoelectric β−phase crystal [21,22]. Meanwhile, the possible biotoxicity and high cost due to large amounts of F elements in the backbone have limited further applications. Another flexible piezoelectric material attracting attention is poly(L−lactide) (PLLA) due to its low cost and biocompatibility. The permanent dipole of the C–O bond in PLLA eliminates the need for electrical poling treatment [23], but its relatively weak piezoelectricity cannot provide sufficient output for sound energy harvesting. Mature strategies for improving the piezoelectric output include co−polymerization [24,25], the use of additives [26,27], and the application of nanostructures [28,29]. Among these methods, the use of additives is a simple and effective way to enhance the piezoelectric properties. However, for the case of PLLA, the requirements for additives are not only the improvement of piezoelectricity but also the maintenance of its low cost and biocompatibility.

In this paper, based on our previous work, we prepared a polling−free piezoelectric composite by blending PLLA and Vitamin B_2_ (VB_2_). The addition of biocompatible VB_2_ significantly enhanced the piezoelectric properties of PLLA through hydrogen bonding and nucleation effects and maintained the low cost and good biocompatibility of PLLA/VB_2_ composites. The PLLA/VB_2_ composites present a constant piezoelectric response to sound in the range of 0 to 20 kHz with an accuracy of 0.1% to recognize the specific sound source and energy density of 0.2 W/cm^−3^ by harvesting sound energy. The synchronous realization of sound recognition and energy harvesting by PLLA/VB_2_ provides great potential for ambient energy harvesting and information collection.

## 2. Materials and Methods

### 2.1. Materials

The PLLA (2003D) used in this study was sourced from NatureWorks (Minneapolis, MN, USA), while VB_2_ and chloroform were procured from Shanghai Aladdin Biochemical Technology Co., Ltd. (Chengdu, China).

### 2.2. Preparation of PLLA/VB_2_ Composites

The PLLA/VB_2_ composites are prepared by dissolving VB_2_ and PLLA in chloroform and stirring for 8 h at 25 °C. The content of VB_2_ is varied from 0 wt% to 20 wt% to PLLA. The target solution was coated with a copper film to form a film with a thickness of 20 μm. The film was then dried under vacuum at 25 °C and annealed at 140 °C. Subsequently, a Ag electrode was coated onto the PLLA/VB_2_ film. Typically, the PLLA/VB_2_ composites with 15 wt% VB_2_ was named PLLA/VB_2_−15.

### 2.3. Characterization of PLLA/VB_2_ Composites

The Fourier transform infrared spectroscopy (FTIR) was recorded on a Bruker Tensor 27 spectrometer with a range from 4000 cm^−1^ to 500 cm^−1^ and a resolution of 4 cm^−1^. The X−ray diffraction (XRD) experiments were carried out on a D/Max2500 VB2t/PC X−ray diffractometer (Rigaku, Tokyo, Japan) with a 2θ range of 5–50°. The differential scanning calorimetry (DSC) curves are recorded on Mettler Toledo DSC 3 instruments (TA Instruments, Shanghai, China) under a N_2_ atmosphere and a heating speed of 10 °C/min. The piezoelectric signals were collected using NI−9238 voltage acquisition (National Instruments, Austin, TX, USA). The cytotoxicity is investigated according to CCK−8 methods.

## 3. Results and Discussion

### 3.1. Characterization of PLLA/VB_2_ Composites

**Figure 1:**
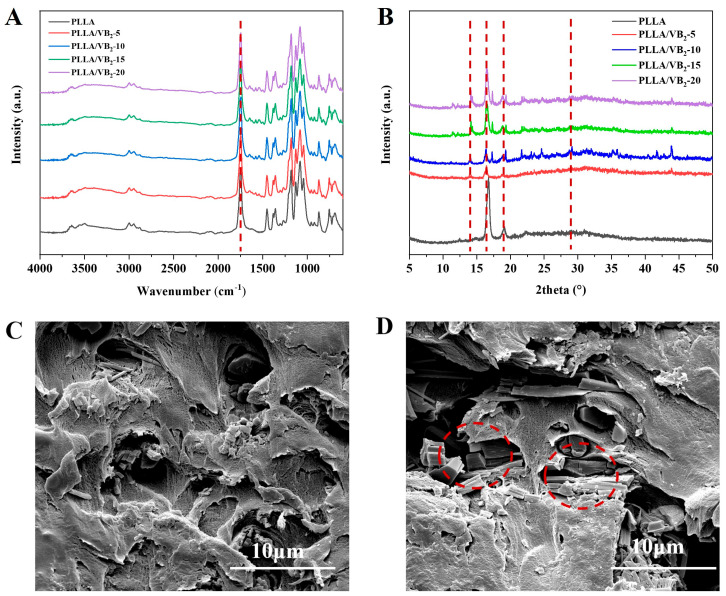
(**A**) FTIR, (**B**) XRD spectra and SEM images of (**C**) PLLA/VB_2_-15, (**D**) PLLA/VB_2_-20.

The FTIR spectra of PLLA/VB_2_ composites are provided in Figure 1A. The vibration of the PLLA ester bond corresponds to the wave number of 1740 cm^−1^. As the VB_2_ content increases, the characteristic peak shifts towards the lower frequency region due to the possible interaction between the C=O dipoles in the ester bond of PLLA and the multiple functional groups including carboxyl, carbonyl, and amino of VB_2_. Furthermore, a broad hydrogen bond peak between 3200 cm^−1^ and 3600 cm^−1^ was observed, suggesting the existence of hydrogen bonds, which simultaneously formed between the PLLA intermolecular chains and between C=O dipoles in PLLA and functional groups in VB_2_. Such hydrogen bond and dipole interaction may promote the orientation and further enhances the piezoelectricity of PLLA/VB_2_.

In Figure 1B, the XRD curves of pure PLLA show several sharp diffraction peaks located at 2θ = 15°, 17°, and 19°, respectively, corresponding to the (010), (110)/(200), and (203) planes in PLLA. The addition of VB_2_ leads to the appearance of a new diffraction peak at 2θ = 30.8°, which belongs to the (003) plane of the β-phase crystal in PLLA [26,27,28]. In addition, with the increasing content of VB_2_, the diffraction peak intensity of the (003) plane increases accordingly. This phenomenon can be attributed to the heterogeneous nucleation promotion of VB_2_ on the crystalline behavior of PLLA, which is a benefit of the improvement of piezoelectricity. Thus, the introduction of VB_2_ not only helps the formation of hydrogen bonds between PLLA and VB_2_, but also promotes the content of polar β-phase crystals. As the hydrogen bond can promote the orientation of C=O dipoles in the easter bonds of PLLA, and the increasing of polar β-phase is benefit to the dipole orientation as reported in the PVDF-based piezoelectric polymer [30], the introduction of VB_2_ can improve the piezoelectricity of PLLA.

Figure 1C,D provides the SEM images of PLLA/VB_2_ composites with different VB_2_ content. The PLLA and VB_2_ are presented as light and dark phases, respectively. VB_2_ distributes uniformly as a rod shape in the PLLA matrix at a content of 15 wt%, while at a higher content (20 wt%) of VB_2_, some agglomerations are observed. Meanwhile, the edge between PLLA and VB_2_ seems indistinct, indicating a strong interaction as evidenced by the hydrogen bond in FITR spectra. 

**Figure 2:**
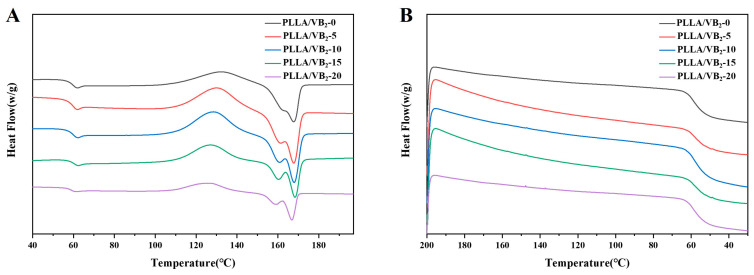
DSC (**A**) heating and (**B**) cooling curves of PLLA/VB_2_.

The DSC heating and cooling curves are provided in Figure 2A,B, respectively. The heating curves show that all the samples have similar thermal transition peaks. The peak at 62 °C is attributed to the glass transition of the samples, and the peak near 125 °C is due to their cold crystallization. The melting peaks of β-crystals and α-crystals are observed at 162 °C and 167 °C, respectively, and these melting peaks are partially overlapped. In addition, the melting enthalpy of β-crystals increased with the increasing VB_2_ content, indicating that the crystallinity of β-crystals in the samples was improved by the addition of VB_2_. Meanwhile, the cold crystallization temperatures of the samples decreased with increasing VB_2_ content, indicating an increase in the crystallinity of the β-crystals and a decrease in the crystallinity of the α-crystals. According to the calculation results, the crystallinity of β-crystals in PLLA increased from 91.93% to 93.97% with increasing VB_2_ content from 5wt% to 15wt%. This phenomenon is attributed to the interaction between the ester bonds in PLLA and the hydroxyl, carbonyl, and amine groups of VB_2_, which is beneficial to promoting the orientation of the PLLA chains and improves the crystallinity of the β-crystals.

### 3.2. Piezoelectric Performance of PLLA/VB_2_ Composites

**Figure 3:**
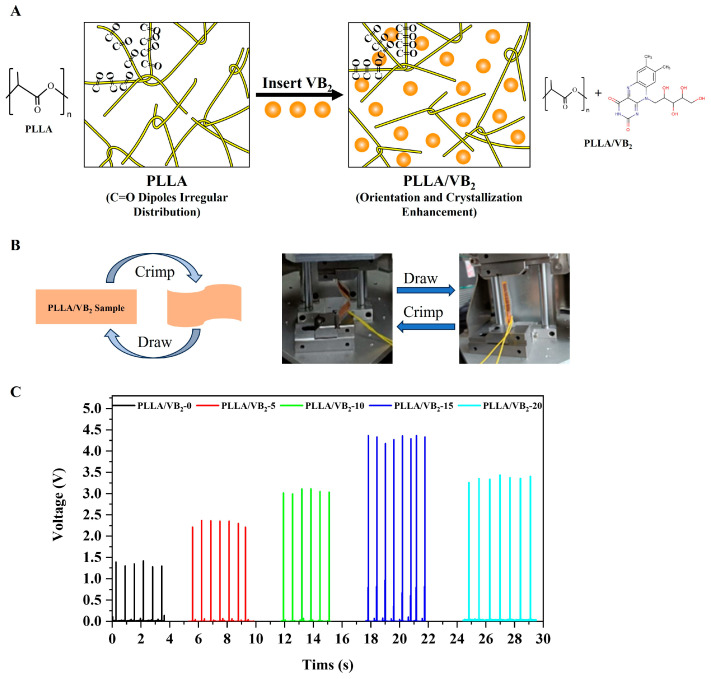
(**A**) Schematic diagram of VB_2_-enhanced PLLA piezoelectric performance, (**B**) schematic, actual diagram platform of output performance of PLLA/VB_2_ composites and (**C**) output voltage of PLLA/VB_2_ nanogenerators with different VB_2_ contents.

The output performances of the PLLA/VB_2_ composites are investigated using a linear motor system to generate a shear force which drives the PLLA/VB_2_ composites to realize a linear reciprocating motion, the electrical signals of PLLA/VB_2_ piezoelectric devices are sampled by voltage acquisition cards (NI-9238). The experimental procedure is shown in Figure 3B, and the output voltages are shown in Figure 3C. The peak voltage of the pure PLLA materials was only 1.45 V. However, as the VB_2_ content in the PLLA/VB_2_ composite increased in the range of 0–15 wt%, the peak voltage of the PLLA/VB_2_-15 composites increased, reaching a maximum output voltage of 4.42 V, which is three times that of the pure PLLA. This increase in output voltage is attributed to the addition of VB_2_ into PLLA, promoting the dipole orientation of the PLLA molecular chains and improving the crystallinity of PLLA, which in turn improves the piezoelectricity of PLLA. However, the peak voltage of the PLLA/VB_2_-20 composites was lower than that of the PLLA/VB_2_-15 composite, which is attributed to the excess content of VB_2_ in PLLA resulting in a non-uniform distribution. Therefore, the PLLA/VB_2_-15 composite seems to have optimum output performance.

In Figure 3A, according to the previous FTIR, XRD and DSC results, the addition of VB_2_ into PLLA leads to the formation of hydrogen bonds and increasing content of β-phase crystal. The hydrogen bonds between the C=O dipoles in PLLA and the function group in VB_2_ may promote the orientation of C=O dipoles [30,31]. Furthermore, the polar β-phase crystal may further improve and maintain the orientation of C=O dipoles. Thus, the piezoelectricity of PLLA is obviously improved.

We compared our PLLA/VB_2_ composites with recent references regarding their sound recognition and energy harvesting performance. Compared to rigid piezoelectric ceramics such as PZT, AlN, and ZnO, our PLLA/VB_2_ material offers several advantages, As shown in Table 1. It is lightweight, flexible, has a low Young’s modulus, and is easy to fabricate and process. Although its piezoelectric coefficient and energy density are lower, PLLA/VB_2_ has the added benefit of reducing noise due to its low speed of sound while converting sound energy into electrical energy [32]. In daily life, low-frequency sound waves primarily exert a shear force on objects. Our PLLA/VB_2_ is sensitive to this shear force due to the piezoelectric constant along the d_14_ direction, making it more prone to vibration or deformation and thus generating electrical energy [33]. Simultaneously, PLLA/VB_2_ possesses the benefits of being non-toxic, naturally degradable, and not requiring polarization, which is absent in traditional rigid piezoelectric ceramics [34,35,36]. As a result, it has a wider potential for large-scale applications in real-world settings.

### 3.3. Piezoelectric Acoustical Performance of PLLA/VB_2_ Composites

**Figure 4:**
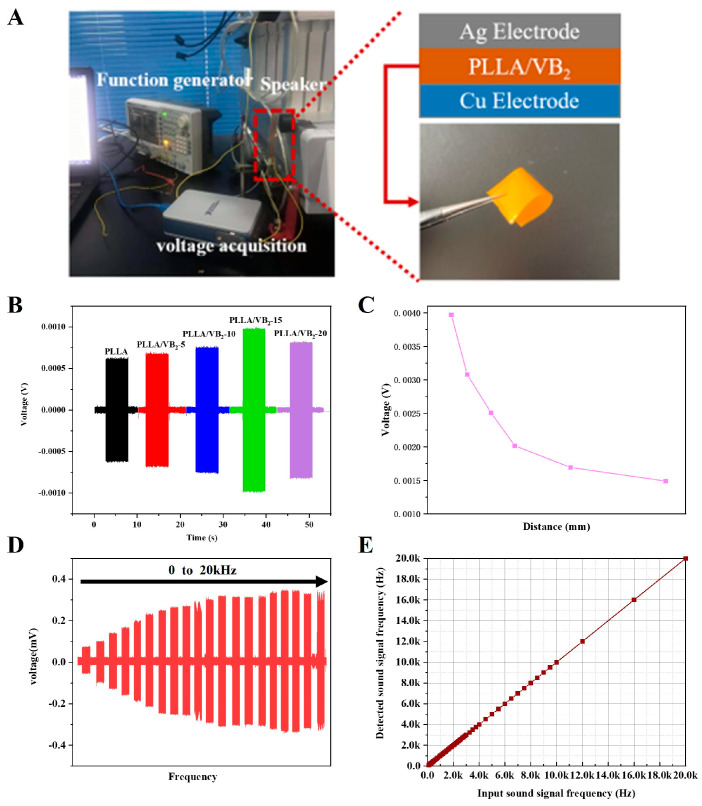
(**A**) Experimental platform for piezoelectric acoustical performance investigation. (**B**) Piezoelectric output of PLLA/VB_2_ composites with different VB_2_ content. (**C**) Piezoelectric output of PLLA/VB_2_-15 under different sound distance. (**D**) Piezoelectric output of PLLA/VB_2_-15 under different sound frequency. (**E**) Measurement error between input and detected sound frequency.

The piezoelectric acoustical response of PLLA/VB_2_ composites is performed using a speaker as a sound source driven by a function generator to play sound samples with different distances and frequencies (as provided in Figure 4A), and the piezoelectric voltage of PLLA/VB_2_ composites is recorded. In Figure 4B, under fixed sound distance and frequency, the PLLA/VB_2_ with increasing VB_2_ content from 0 wt% to 15 wt% shows an increasing piezoelectric output from 0.67 mV to 1.10 mV, benefitting from promotion in the dipole orientation and crystalline formation by the addition of VB_2_. With a higher content of VB_2_ (20 wt%), the piezoelectric output decreases to 0.83 mV, which is due to the excessive content of VB_2_ in PLLA, resulting in the uneven distribution of VB_2_ in PLLA. The maximum piezoelectric output is observed for PLLA/VB_2_-15; thus, it is selected for further investigation on piezoelectric acoustical performance.

Figure 4C,D show the piezoelectric output of PLLA/VB_2_-15 with variated sound distance and frequency. The output voltage of PLLA/VB_2_-15 increases with the decrease in sound distance (which equals to increase in sound amplitude). As sound is one of the mechanical waves, it leads to surface vibration and deformation when it propagates to the surface PLLA/VB_2_-15. Meanwhile, piezoelectric charges are generated in response to the change in the degree of polarization. In this view, shorter distance and higher amplitude intensify this process, leading to higher piezoelectric voltage. Meanwhile, PLLA/VB_2_-15 show higher piezoelectric output to sound with higher frequency ranging from 0 to 20kHz, indicating frequency-dependent properties within the human auditory range.

The frequency-dependent response of PLLA/VB_2_-15 enables its capacity to recognize sound frequency. The voltage–time curves can be converted to amplitude–frequency curves by using a fast Fourier transform algorithm. Thus, a standard sound sample with a fixed frequency is used to investigate the detected error of PLLA/VB_2_-15. In Figure 4E, the PLLA/VB_2_-15 shows an average error of less than 0.1% at a frequency from 0 to 20 kHz, indicating a high accuracy of sound frequency recognition.

### 3.4. Sound Recognition and Energy Harvesting Performance of PLLA/VB_2_ Composites

We asked two students (one male and one female) to say “hello” to the PLLA/VB_2_-15 film samples. Upon acoustic excitation, PLLA/VB_2_-15 generates voltage signals. The voltage acquisition card collects and records the output voltage signal of PLLA/VB_2_-15.

**Figure 5:**
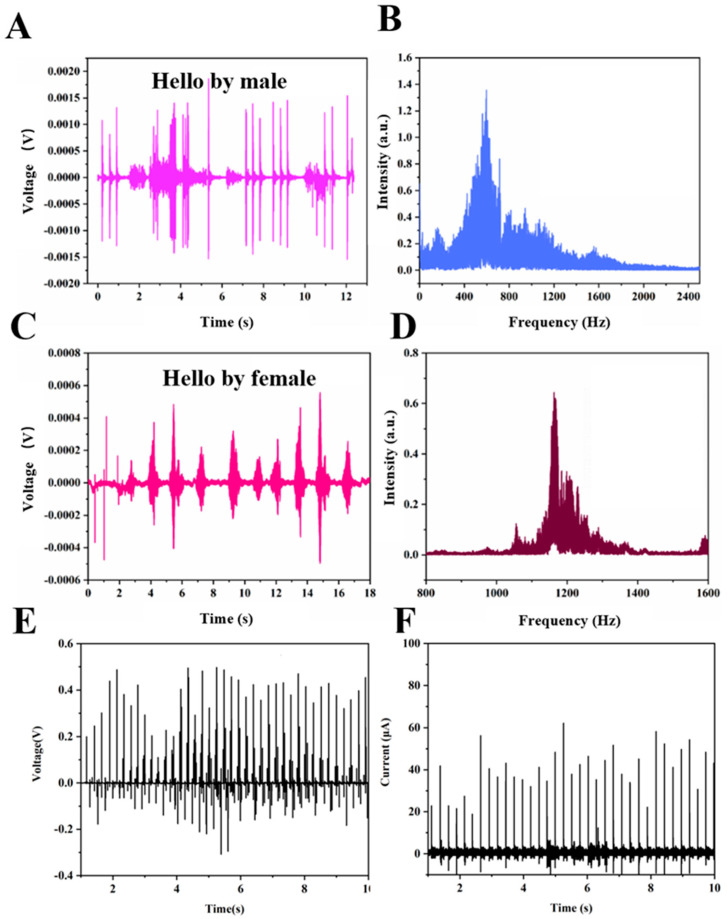
Piezoelectric output of PLLA/VB_2_-15 stimulated by (**A**) male and (**C**) female sound sample and (**B**,**D**) FFT curves; (**E**) piezoelectric voltage and (**F**) current of PLLA/VB_2_-15 stimulated by sound sample.

The sound samples of the male and female saying “hello” are used to investigate the sound recognition performances of PLLA/VB_2_-15 composites. According to the FFT curves (Figure 5C,D), which were converted from the voltage–time curves (Figure 5A,C), the frequency of male voice samples is mainly concentrated in the low-frequency region from 400 to 800 Hz, while that of the female voice samples is concentrated in the high-frequency region from 1000 to 1400 Hz. By comparing and analyzing the frequency spectral characteristics of voice, we can achieve sound recognition of certain keywords or specific vocabulary. Additionally, the amplitude and distance of the sound sample are fixed at 50 db and 30 cm, respectively, to evaluate the energy harvesting performances of PLLA/VB_2_-15 composites. By recording the piezoelectric voltage and current in Figure 5E,F, the average piezoelectric voltage and current are 0.25 V and 30 μA, respectively. Thus, the energy density of the PLLA/VB_2_-15 composite is calculated to be 0.2 W/cm^−3^. With the help of a rectifying circuit, this device can drive low-power devices such as LEDs (Figure S1).

In our daily lives, sound recognition technology is used to judge or distinguish the speaker by capturing the speaker’s voice and extracting the voiceprint feature signal. Since the PLLA/VB_2_-15 piezoelectric device has good sound recognition capability, it is used as an acoustic sensor. 

**Figure 6:**
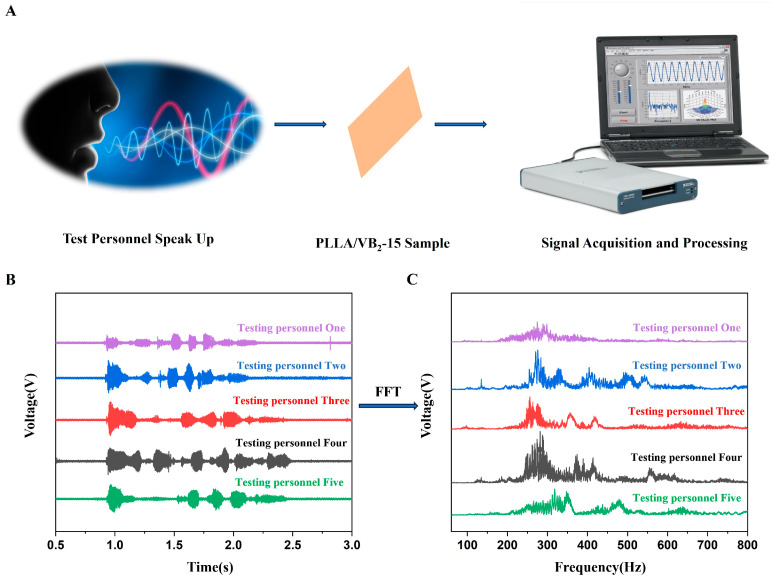
(**A**) Schematic diagram of acoustic device testing, (**B**) voltage–time curves and (**C**) intensity–frequency curves of PLLA/VB_2_-15 acoustic sensor.

Five different people successively shouted the same seven Chinese characters based on their own speaking habits when they were 40 cm away from the PLLA/VB_2_-15 acoustic sensor, and the PLLA/VB_2_-15 acoustic sensor generated piezoelectric voltage under the sound stimulations. The piezoelectric voltage of the PLLA/VB_2_-15 acoustic sensor was recorded by the voltage acquisition card. The experimental procedure is shown in Figure 6A. In Figure 6B, the performance of the time interval of spitting words is different and the intensity of output voltage corresponding to each word is also different. The speaker can be roughly distinguished based on their speaking frequency and loudness characteristics. 

Considering the variable speaking frequency and loudness when people are in different surroundings, the FFT algorithm is employed to further improve the recognition accuracy of the PLLA/VB_2_-15 acoustic sensor. The FFT algorithm can convert the voltage–time curves from PLLA/VB_2_-15 acoustic sensor to intensity–frequency curves, which may help to better distinguish the speakers. As shown in Figure 6C, differences in the frequency of the same word spoken by each student were found because the frequency of each person’s articulation is based on the characteristics of their vocal cords. These extracting the characteristic information of pronunciation frequency from the frequency domain signal can further distinguish the speakers.

### 3.5. Cytotoxicity and Biocompatibility of PLLA/VB_2_ Composites

CCK-8 (Cell Counting Kit-8) is a commonly used cell counting kit for evaluating cell viability and proliferation [39]. CCK-8 is widely used in cell biology research, pharmacology research, and drug screening because it is easy to use, does not require the handling of toxic solutions, and the test results can be measured directly on a microplate reader [40].

**Figure 7:**
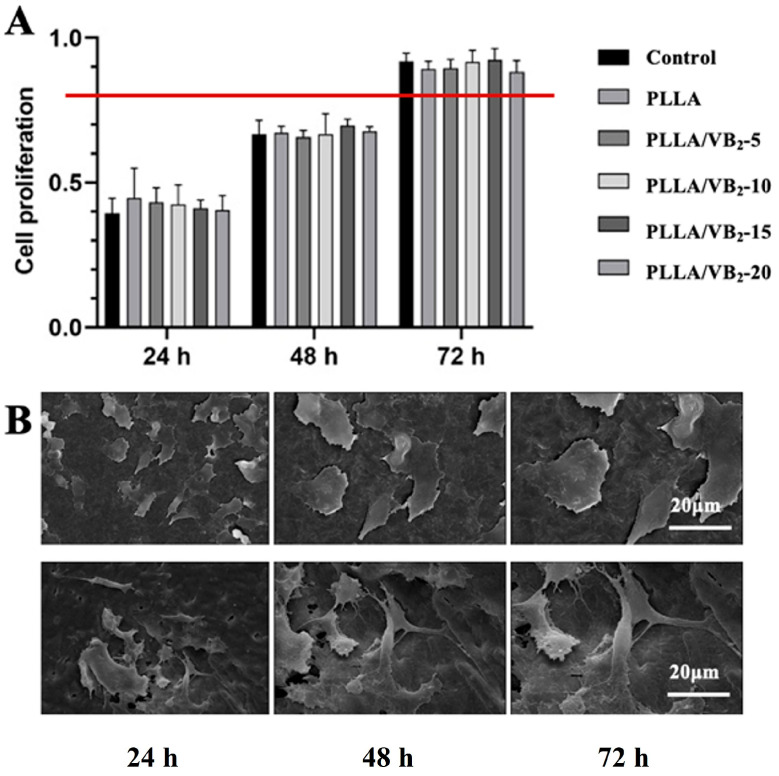
(**A**) The cytotoxicity of PLLA/VB_2_ composites and cell morphology at different incubating time and (**B**) rod and shuttle cell morphology.

The CCK-8 methods are used to evaluate the possible cytotoxicity of PLLA/VB_2_ composites. With increasing incubating time up to 72 h, the relative growth rate of PLLA/VB_2_ composites (Figure 7A) is higher than 80%, which belongs to “grade 0”, regarded as no cytotoxicity. In Figure 7B, the incubated cells present various shapes such as rod and shuttle, indicating they grow in good conditions. Thus, the PLLA/VB_2_ composites present good biocompatibility and no cytotoxicity.

## 4. Conclusions

In summary, the poling-free and flexible PLLA/VB_2_ composites are prepared to simultaneously realize sound recognition and energy harvesting. Through the promotion of dipole orientation and crystalline formation by the addition of VB_2_, the piezoelectric performances are significantly improved. The PLLA/VB_2_ composites can accurately detect the sound frequency with an error of less than 0.1%, which enables them to recognize specific sound samples. Meanwhile, the PLLA/VB_2_ composites present an energy density of 0.2 W/cm^-3^ to power up LEDs. The capacity to recognize frequency and harvest energy from sound makes PLLA/VB_2_ composites great value for potable energy supplies and information collections.

## Figures and Tables

**Table 1:** Comparative data between PLLA/VB_2_ and other piezoelectric materials.

Performance Index	Units	PZT [35]	AlN [37]	ZnO [38]	PLLA/VB_2_
Density	(kg/m^3^)	7800	3230	5610	1250
Young’s modulus Y	(GPa)	60	308	201	2.7
Dielectric constant εr	ε_r_33	2400	10.5	11	2.6
Piezoelectric constant	(pC/N)	d_33_ = 500	d_33_ = 5.5	d_33_ = 12.4	d_14_ = 10
Sound velocity	m/s	6500	10000	5500	2300
Whether polarization is required	Yes or No	Yes	Yes	Yes	No
Whether it is toxic or not	Yes or No	Yes	Yes	Yes	No

## Data Availability

Data are contained within the article.

## Supplementary Materials

The following supporting information can be downloaded at: https://www.mdpi.com/article/10.3390/polym16081071/s1, Figure S1: Powering up of LED by energy harvesting of PLLA/VB_2_-15. Figure S2: PLLA/VB_2_-15 Response of a sound transducer subjected to excitation by seven fundamental scales (Do, Re, Mi, Fa, So, La, Si). (a) time-domain response; (b) frequency-domain response.

## References

[1] Roundy S., Wright P.K., Rabaey J. (2003). A study of low level vibrations as a power source for wireless sensor nodes. Comput. Commun..

[2] Guerreiro A.J., Pereira C.L., Fernandes J.C.S., Fernandes F.M. (2019). Energy harvesting for wearable devices: A review. Sensors.

[3] Wang W., Yang X., Zhu Y., Wu Z., Liu Y., Wang Z.L. (2007). Nanogenerator: A hybrid energy harvesting cell with multiple sources. Nanotechnology.

[4] Sodano H.A., Inman D.J. (2007). Experimental comparison of piezoelectric energy harvesting circuits for powering wireless sensors. Smart Mater. Struct..

[5] Topalov E., Stoychev I.G. (2014). Piezoelectric energy harvesting: A review. Izv. Vis. Ucheb. Zaved. Elektromekh..

[6] Aktakka A., Senentxu Lanceros-Méndez M.P. (2019). Energy harvesting solutions for wearable technologies. Materials.

[7] Mirvakili S.M., Hajjaj M., Tutumluer S., Wang L., Beeby S.P. (2019). Development of wearable multi-modal energy harvesting systems for IoT applications. Appl. Energy.

[8] Park J.Y., Son S.H., Kim J.P. (2015). A review of energy harvesting for portable electronics. Asian J. Chem..

[9] Xiao L., Chan H.L.W., Wang X. (2008). Piezoelectric energy harvesting under different temperature and loadings. Smart Mater. Struct..

[10] Zhang L., Xu Z., Li Y., Chen H.Y. (2020). Piezoelectric nanogenerators for self-powered sensing, self-powered communication and self-powered healthcare. Nano Energy.

[11] Kim H., Ghaffari R., Kim Y.S., Lee H.J., Kim H.R., Kim S.M., Choi Y.D., Chung C.S., Kim J.K. (2011). Stretchable, transparent graphene interconnects for arrays of microscale inorganic light emitting diodes on rubber substrates. Nano Lett..

[12] O’Keefe S.R., Ehlert J.C., Cross L.E., Weigand P.M. (1973). Use of bimorph elements for efficient piezoelectric energy conversion. J. Appl. Phys..

[13] Roundy S., Wright P.K. (2005). Energy scavenging for wireless sensor networks: With special focus on vibrations. Proc. IEEE.

[14] Sun W., Kou F., Wang J.J., Wang X., Yang Q., Zhou F., Li J., Xu S., Zhang J. (2016). Toward a self-powered wireless temperature sensor system based on a piezoelectric energy harvester using a cantilever beam. IEEE Sens. J..

[15] Wang X., Lv F., Xiang Y., Zhang Y., Hao Z., Yan C., Su J. (2019). A self-powered and low-cost acoustic sensor based on a piezoelectric energy harvester for wireless structural health monitoring. Front. Built Environ..

[16] Yang B., Hahn J.M., Yang Y., Kim E., Kim B.W., Sung H.J., Lee S. (2013). An energy harvesting device using a bistable composite laminated beam for vibration-based wireless sensor nodes. J. Intell. Mater. Syst. Struct..

[17] Zhang X., Ni Y., Sun Y., Shi J. (2013). A vibration energy harvesting device with magnetoelectric laminate composites. Appl. Phys. Lett..

[18] Zhang W., Alamry M.Y., ElAtab H.A., Irazoqui J.P. (2016). Vibration-based energy harvesting from a wearable bracelet using a piezoelectric double-clamped beam. Smart Mater. Struct..

[19] Lee N., Shin J., Hong J., Zou X., Yu W., Lee D.Y. (2018). Fabric-based piezoelectric energy harvesting device based on polyvinylidene fluoride-co-trifluoroethylene: A feasibility study. Sensors.

[20] Liu Z., Cheng G., Xu L., Kang Z. (2010). A piezoelectric nanogenerator based on PVDF-TrFE thick films for harvesting vibration energy. J. Micromech. Microeng..

[21] Hu Z., Qi Y., Li Z., Zhang M., Lu F., Guo X. (2013). Enhancing the β-phase crystallinity of PVDF for integration of PVDF and PS on a single chip. Sens. Actuators A Phys..

[22] Li Z., Kim C.Y., Vashaee D. (2015). Enhancement of piezoelectric properties of PVDF-TrFE through Cr doping. J. Micromech. Microeng..

[23] Sodano H.A., Park G., Inman D.J. (2005). Comparison of polyvinylidene fluoride and lead zirconate titanate in an energy harvesting vibration-based device. Smart Mater. Struct..

[24] Kim S., Hong S., Lee S., Lee M. (2011). Highly piezoelectric poly(vinylidenefluoride-co-trifluoroethylene) random copolymer with unusual crystal phases. Macromolecules.

[25] Shrout T.R., Jitianu S., Goeringer R.L., Cann D.P., Subramanian M.A., Sharma N.K., Shaw T.M. (1999). Piezoelectric property enhancement of poly(vinylidene fluoride) with inclusion of BaTiO_3_ ceramics. J. Electroceram..

[26] Xu C., Song Y., Han M., Zhang H. (2021). Portable and wearable self-powered systems based on emerging energy harvesting technology. Microsyst. Nanoeng..

[27] Yuan D., Chen H., Zhang X. (2014). Enhancing piezoelectric response of poly(vinylidene fluoride) by incorporating reduced graphene oxide. Carbon.

[28] Wang J., Cai S., Yao M., Li Y., Tang J., Li P. (2014). Enhanced piezoelectric performance of PVDF/ZnO nanorod composite films by the bridging effect of carbon nanotubes. J. Mater. Sci..

[29] Yang J., Fan X., Xiang Y., Wang Y., Gong L., Yu K., Gao B., Su J. (2016). Preparation and piezoelectricity of poly(vinylidene fluoride-co-trifluoroethylene) nanocomposites with BaTiO_3_ nanocrystals decorated graphene oxide. Appl. Phys. Lett..

[30] Sumanta K.K., Sandip M., Anand K.A., Amit K.D., Anirban M., Sarbaranjan P., Aswini B., Ranadip B., Lopamudra H., Avnish K.M. (2019). Designing high energy conversion efficient bio-inspired vitamin assisted single-structured based self-powered piezoelectric/wind/acoustic multi-energy harvester with remarkable power density. Nano Energy.

[31] Akiko O., Toshiki H., Takashi W., Yoshio O., Toshihiro K. (2010). Electrospun microfiber meshes of silicon-doped vaterite/poly(lactic acid) hybrid for guided bone regeneration. Acta Biomater..

[32] Duan B., Wu K., Chen X., Ni J., Ma X., Meng W., Lam K.H., Yu P. (2023). Bioinspired PVDF Piezoelectric Generator for Harvesting Multi-Frequency Sound Energy. Adv. Electron. Mater..

[33] Zin S.H.M., Velayutham T.S., Furukawa T., Kodama H., Gan W.C., Chio-Srichan S., Kriechbaum M., Nakajima T. (2022). Quantitative study on the face shear piezoelectricity and its relaxation in uniaxially-drawn and annealed poly-l-lactic acid. Polymer.

[34] Ramadan K.S., Sameoto D., Evoy S. (2014). A review of piezoelectric polymers as functional materials for electromechanical transducers. Smart Mater. Struct..

[35] Panda P.K., Sahoo B. (2015). PZT to Lead Free Piezo Ceramics: A Review. Ferroelectrics.

[36] Fu Y.Q., Luo J.K., Nguyen N.T., Walton A.J., Flewitt A.J., Zu X.T., Li Y., McHale G., Matthews A., Iborra E. (2017). Advances in piezoelectric thin films for acoustic biosensors, acoustofluidics and lab-on-chip applications. Prog. Mater. Sci..

[37] Marauska S., Hrkac V., Dankwort T., Jahns R., Quenzer H.J., Knöchel R., Kienle L., Wagner B. (2012). Sputtered thin film piezoelectric aluminum nitride as a functional MEMS material. Microsyst. Technol..

[38] Crisler D.F., Cupal J.J., Moore A.R. (1968). Dielectric, piezoelectric, and electromechanical coupling constants of zinc oxide crystals. Proc. IEEE.

[39] Cai L., Qin X., Xu Z., Song Y., Jiang H., Wu Y., Ruan H., Chen J. (2019). Comparison of Cytotoxicity Evaluation of Anticancer Drugs between Real-Time Cell Analysis and CCK-8 Method. ACS Publ..

[40] Liao J., Zheng H., Fei Z., Lu B., Zheng H., Li D., Xiong X., Yi Y. (2018). Tumor-targeting and pH-responsive nanoparticles from hyaluronic acid for the enhanced delivery of doxorubicin. Int. J. Biol. Macromol..

