# Supplementary material for: Synchronous Sound Recognition and Energy Harvesting by Flexible Piezoelectric PLLA/VB2 Composites
Source: Polymers (Basel). 2024 Apr 11;16(8):1071. doi: 10.3390/polym16081071 (PMC11053639; doi:10.3390/polym16081071)
Supplement: Supplementary file 1 [file polymers-16-01071-s001.zip › polymers-2934521-supplementary.pdf]

**Synchronous Sound Recognition and Energy Harvesting by Flexible Piezoelectric PLLA/$VB_2$ Composites**

## Supporting Information

*Qian Zhang*[1], *Qiang Liu*[1], *Weidong Xue*[1], *Yong Xiang*[1], *Xiaoran Hu*[1, *]

[1] School of Materials and Energy, University of Electronic Science and Technology of China, Chengdu, China

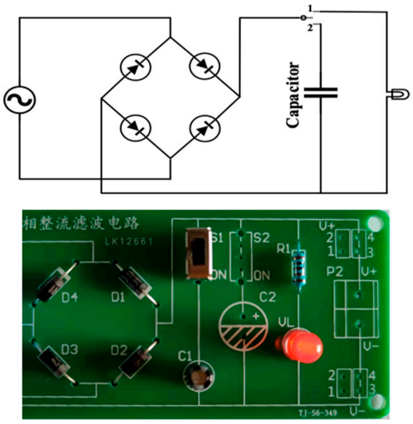


**Figure S1.** Powering up of LED by energy harvesting of PLLA/$VB_2$-15

Seven basic scale samples (Do, Re, Mi, Fa, So, La, Si) were selected to be sounded by a computer-controlled voice system, which excited the PLLA/$VB_2$-15 transducer to generate voltage signals, which were sampled by a voltage acquisition card and transmitted to the EAQ-express host computer for display and recording, the results of the experiment are shown in Figure S2(A). As shown in Figure S2(B), the time-domain signals of the seven basic scales were processed by the FFT of the EAQ-express software to obtain the frequency-domain signals (Do: 262.6 Hz, Re: 294.5 Hz, Mi: 329.7 Hz, Fa: 349.2 Hz, So: 392.1 Hz, La: 494.5 Hz, Si: 523.3 Hz), and the seven basic scales corresponded to different frequencies (Do: 262.7 Hz, Re: 294.8 Hz, Mi: 330.1 Hz, Fa: 349.1 Hz, 394.1 Hz, 330.1 Hz, 330.1 Hz, 330.1 Hz). Hz), 7 basic scales correspond to different frequencies (Do: 262.7 Hz, Re: 294.8 Hz, Mi: 330.1 Hz, Fa: 349.6 Hz, So: 392.7 Hz, La: 494.5 Hz, Si: 523.5 Hz). The frequency domain signals were compared with the standard 7 fundamental scale frequencies, and the relative error of each scale frequency was within 1%. Therefore, this experiment shows that PLLA/$VB_2$-15 can be used as a high-performance synchronized speech recognition device.

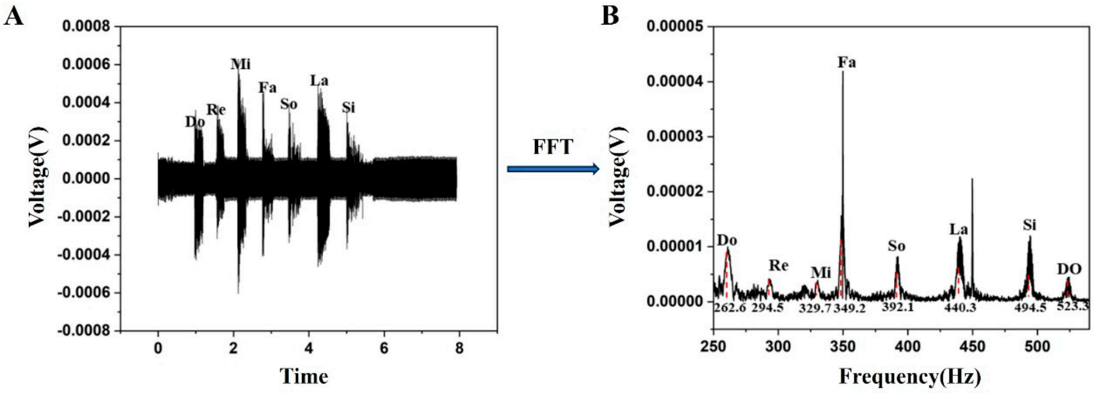


**Figure S2.** PLLA/$VB_2$-15 Response of a sound transducer subjected to excitation by seven fundamental scales (Do, Re, Mi, Fa, So, La, Si). (a) time-domain response; (b) frequency-domain response
